# Public Engagement in Health Policy‐Making for Older Adults: A Systematic Search and Scoping Review

**DOI:** 10.1111/hex.70008

**Published:** 2024-08-26

**Authors:** Jeonghwa You, Rebecca Ganann, Michael Wilson, Soo Chan Carusone, Maggie MacNeil, Carly Whitmore, Andrea Dafel, Roma Dhamanaskar, Eugenia Ling, Lance Dingman, A. Tina Falbo, Michael Kirk, Joyce Luyckx, Penelope Petrie, Donna Weldon, Katherine Boothe, Julia Abelson

**Affiliations:** ^1^ Department of Health Research Methods, Evidence and Impact (HEI) McMaster University Hamilton Canada; ^2^ School of Nursing McMaster University Hamilton Canada; ^3^ Department of Health Research Methods, Evidence and Impact McMaster University Hamilton Canada; ^4^ McMaster Health Forum Hamilton Canada; ^5^ McMaster Collaborative for Health and Aging Hamilton Canada; ^6^ Department of Political Science McMaster University Hamilton Canada; ^7^ Centre for Health Economics and Policy Analysis (CHEPA) McMaster University Hamilton Canada

**Keywords:** health policy, long‐term care, older adults, public engagement, public involvement, scoping review, senior

## Abstract

**Introduction:**

As the world's population ages, there has been increasing attention to developing health policies to support older adults. Engaging older adults in policy‐making is one way to ensure that policy decisions align with their needs and priorities. However, ageist stereotypes often underestimate older adults' ability to participate in such initiatives. This scoping review aims to describe the characteristics and impacts of public engagement initiatives designed to help inform health policy‐making for older adults.

**Methods:**

A systematic search of peer‐reviewed and grey literature (English only) describing public engagement initiatives in health policy‐making for older adults was conducted using six electronic databases, Google and the Participedia website. No geographical, methodological or time restrictions were applied to the search. Eligibility criteria were purposefully broad to capture a wide array of relevant engagement initiatives. The outcomes of interest included participants, engagement methods and reported impacts.

**Results:**

This review included 38 papers. The majority of public engagement initiatives were funded or initiated by governments or government agencies as a formal activity to address policy issues, compared to initiatives without a clear link to a specific policy‐making process (e.g., research projects). While most initiatives engaged older adults as target participants, there was limited reporting on efforts to achieve participant diversity. Consultation‐type engagement activities were most prevalent, compared to deliberative and collaborative approaches. Impacts of public engagement were frequently reported without formal evaluations. Notably, a few articles reported negative impacts of such initiatives.

**Conclusion:**

This review describes how public engagement practices have been conducted to help inform health policy‐making for older adults and the documented impacts. The findings can assist policymakers, government staff, researchers and seniors' advocates in supporting the design and execution of public engagement initiatives in this policy sector.

**Patient or Public Contribution:**

Older adult partners from the McMaster University Collaborative for Health and Aging provided strategic advice throughout the key phases of this review, including developing a review protocol, data charting and synthesis and interpreting and presenting the review findings. This collaborative partnership was an essential aspect of this review, enhancing its relevance and meaningfulness for older adults.

## 1 Introduction

Older adults are one of the largest user groups of healthcare services, and their health needs are increasingly diverse and complex [1]. Public engagement (hereafter PE) refers to the practice of involving the public in different stages of policy‐making, which is distinguished from the traditional governance model, where policymakers, government, officials and experts are engaged in policy‐making without additional public input [2]. PE initiatives are believed to result in health policies and programmes that are responsive to the needs of older adults, in turn, leading to better health outcomes [3]. In this regard, engaging with older adults is an important vehicle for ensuring that their perspectives are taken into account in policy‐making processes.

Yet, older adults are often excluded from or are given few opportunities to participate in policy‐making processes that are designed for them [4]. Several factors may contribute to the exclusion of older adults from PE initiatives. Physical and cognitive frailty are often assumed to be barriers as they may limit their willingness to participate and understand complicated policy information therefore limiting meaningful engagement [5]. In addition, ageist stereotypes and attitudes, social isolation and the lack of access to digital technologies and skills that are necessary for participation in virtual PE initiatives can limit older adults' opportunities to participate in policy‐making processes, leading to their exclusion from these processes [4, 6].

In this context, PE organizers often engage with community organizations and other civil society groups in health policy‐making for older adults instead of directly involving older adults themselves [7]. Such organizations serve as a channel for eliciting the views of older adults as they often possess extensive knowledge of the needs and preferences of older adults [5, 6]. In such cases, PE initiatives for policy‐making related to older adults are expected to have unique characteristics that differ from standard engagement practices, where policy recipients directly share their input [8]. Despite these assumed differences, there is currently no evidence synthesis that examines characteristics of PE initiatives specifically designed for informing policy‐making for older adults. Since the effectiveness of PE is influenced by design factors such as the selection of participants and the tools and resources used to facilitate engagement activities [4], it is important to understand how PE is implemented in healthcare policy‐making for older adults.

We aimed to identify PE initiatives from existing literature that were designed to inform system‐level health policy‐making for older adults, to provide an overview of the literature available in this area and to describe the key characteristics and impacts of these initiatives. The significance of this review lies in its exploration of the distinctive characteristics of PE, with the goal of providing insights into the design and conduct of PE practices within the specific context of health policy‐making for older adults.

## 2 Methods

We used a scoping review design which included the identification of research questions and relevant studies, the selection of studies, data charting and the summary and presentation of results [9]. Our scoping review followed the Preferred Reporting Items for Systematic Reviews and Meta‐analyses (PRISMA) Extension for Scoping Reviews guidelines [10].

### 2.1 Information Sources and Search Strategy

The search strategy was developed by the lead author in collaboration with a university librarian (see Supporting Information S1: Appendix 1 for the full search strategies). In the spring of 2022, we searched six databases (MEDLINE, CINAHL, Politics Collection, HealthStar, Social Science Citation Index and AgeLine) for both peer‐reviewed articles and grey literature, without any geographical, methodological or time restrictions; only articles reported in English were included. Additional searches were conducted in Google Advanced and Participedia, an online collaborative knowledge base for PE, participatory governance and democratic innovations [11].

### 2.2 Eligibility Criteria

Eligibility criteria were purposefully broad to capture a wide array of PE initiatives. Inclusion and exclusion criteria were informed by our interest in examining PE initiatives that lay at the intersection of three areas: PE, system‐level policy‐making and health policies for older adults (see Table 1 for details).

**Table 1:** Description of eligibility criteria.

Inclusion criteria	Exclusion criteria
Public engagement (PE): PE involves any form of deliberately engaging the public in relation to policy‐making, organized by some entities. Engagement takes a variety of forms and encompasses various activities and roles played by the public in different health systems and policy‐making stages. The public includes older adults, their family and friends, representative organizations advocating for their rights (e.g., charities and volunteer groups) and the general public. Engagement may be one‐off or ongoing activities [2].	Engagement focusing on professionals only Grass‐roots or bottom‐up engagement movements Non‐empirical or insufficient empirical evidence about PE
Policy‐making at the system level: Articles dealing with policy‐making within health systems were included. A well‐recognized taxonomy of governance, financial and delivery arrangements within health systems [12] was used to consistently operationalize this criterion.	Individual healthcare decisions Organizational contexts Community settings
Health policies for older adults: Health policies for older adults covering various topics, health conditions and populations were included.	Policies indirectly affecting health (e.g., social policies)

### 2.3 Article Selection

All literature retrieved from the electronic databases was uploaded to Covidence software. After removing duplicates, team members were randomly paired into review teams (J.Y., C.W., S.C.C., M.M., R.G. and J.A.) and independently screened the titles and abstracts of retrieved papers to assess eligibility. Any papers considered relevant by both reviewers were included for full‐text review. Full‐text papers were then independently screened by randomly paired reviewers (J.Y., C.W., R.D., R.G., E.L., A.D., S.C.C. and M.M.). For both screening stages, disagreements between the reviewers were discussed among themselves; any remaining disagreements were resolved by a third reviewer.

The lead author screened Google and Participedia search results using the titles, abstracts, summaries or contents (whichever was available). Google searches were screened for the first 10 pages, with an additional five pages if relevant materials were found. The search was ended when no more relevant information was found. All search results on Participedia underwent full‐text screening. All documents considered relevant were entered into an Excel sheet, and duplicates were removed. The lead author (J.Y.) reviewed the full text of all articles. A second reviewer (J.A.) screened 30% of the entries. The inter‐rater agreement was close to unanimous, so no further double‐reviewing of additional papers was deemed necessary.

### 2.4 Data Charting

A structured form for data charting was developed by the first and senior authors (J.Y. and J.A.) and was pilot‐tested by eight members (J.Y., A.D., C.W., E.L., M.M., R.G., R.D. and S.C.C.). Based on team members' feedback, the form was revised to incorporate the definition of participant diversity using the PROGRESS‐Plus framework [13] and exclude the items related to policy‐making stages. Final data items to be extracted include basic information about included articles (e.g., author and publication year) and PE‐relevant data (e.g., participants, participant diversity, inclusion strategy, engagement activities and impacts).

Both predefined categories and open‐text boxes were used to chart information, including engagement types and reported impacts. The categories for engagement types were derived from a well‐recognized typology [14] selected for its perceived usability in capturing various types of engagement initiatives in system‐level policy‐making. The categories were defined as follows: ‘share’ (one‐way communication where PE organizers provide information to participants to help them understand policy issues), ‘consult’ (PE organizers gather feedback from participants on proposed programmes or policies), ‘deliberate’ (participants carefully consider policy options based on available information and engage in discussions to recommend a policy solution) and ‘collaborate’ (participants work with other stakeholders to address an issue and develop and apply solutions for policy issues, often integrated into organizational governance structures). Similarly, categories for PE impacts were drawn from a report that identified commonly reported engagement impacts based on literature reviews [15]. The impacts were categorized into ‘instrumental’ (PE improves policy‐making in terms of its processes and/or outcomes), ‘intrinsic’ (PE in policy‐making is a value in itself as it promotes a healthier democracy and enhances fairness and justice), ‘developmental’ impacts (participants develops new or improves existing skills or knowledge as a result of their engagement) and ‘others’ [15].

Random pairs of reviewers independently extracted the data from each article (J.Y., A.D., C.W., E.L., M.M., R.G., R.D. and S.C.C.). The results were compared, and any discrepancies were resolved by consensus, with a third reviewer resolving any remaining disagreements.

### 2.5 Quality Appraisal

While quality appraisal is generally considered an optional component for a scoping review, we conducted quality appraisal for all included articles as part of our objective of describing the impacts of PE initiatives. Given that robust and comprehensive evaluations of PE impacts are often lacking [16], we sought to gain insight into the methodological rigour of this study element, facilitating a more informed interpretation of the findings regarding PE impacts.

Using the MMAT Mixed Methods Appraisal Tool, quantitative, qualitative and mixed‐methods studies were appraised [17]. The AACODS checklist (authority, accuracy, coverage, objectivity, date, significance) was also used to appraise grey literature [18]. As calculating an overall score based on criteria ratings was discouraged for better reporting of the qualities [17], the appraisal results were presented through a description of specific criteria that received poor ratings. Quality assessment of each article was independently conducted by randomly paired reviewers (J.Y., A.D., C.W., E.L., M.M., R.G., R.D. and S.C.C.), and any disagreements were resolved by consensus.

### 2.6 Data Analysis and Synthesis

All articles meeting the inclusion criteria were analysed regardless of their quality appraisal results. Extracted data were analysed and synthesized descriptively without interpreting beyond what was explicitly stated in the original studies [19]. We used existing typologies or frameworks to consistently categorize key concepts. The Health Systems Evidence (HSE) taxonomy was employed to categorize health system arrangements [12], and the PROGRESS‐Plus was utilized to categorize participant diversity [13]. Additionally, we utilized Health Quality Ontario's Patient Partnering Framework to categorize types of engagement activities [14]. The categories of PE impacts were adopted based on the concepts of PE benefits in healthcare policy [15].

### 2.7 Collaboration With Older Adults

We collaborated with a total of nine older adult partners from the McMaster University Collaborative for Health and Aging (L.D., A.T.F., M.K., J.L., P.P., D.W., and three other members), aimed at supporting and building capacity for patient‐oriented research in the field of ageing. The partners were recruited before the initiation of this scoping review. The collaboration occurred over the key phases of this review via three online meetings and email correspondence. Before each meeting, meeting materials were shared for review. During the meetings, the lead author presented the agenda and led discussions, while a co‐author (S.C.C.), closely with the partners, facilitated the meetings. Follow‐up emails were sent to provide any requested information after the meetings. The partners contributed to the research proposal by reviewing and confirming the search strategy and providing suggestions for additional information to be charted (e.g., age of participants who were considered older adults). After completing data charting, the partners identified areas for further exploration based on preliminary results. The lead author then delved into these areas and incorporated them when their suggestions were identifiable from the results (e.g., check and report on how the reported impacts were assessed). After data analysis and synthesis, the partners provided feedback on the summary, significantly enriching the direction of the discussion section. Additionally, they reviewed drafts of a research brief and publication manuscript and offered feedback on policy recommendations and other design elements.

This collaboration was an essential aspect of this review, ensuring that the research was relevant and meaningful for older adults in addition to the field of health policy engagement.

## 3 Results

**Figure 1:**
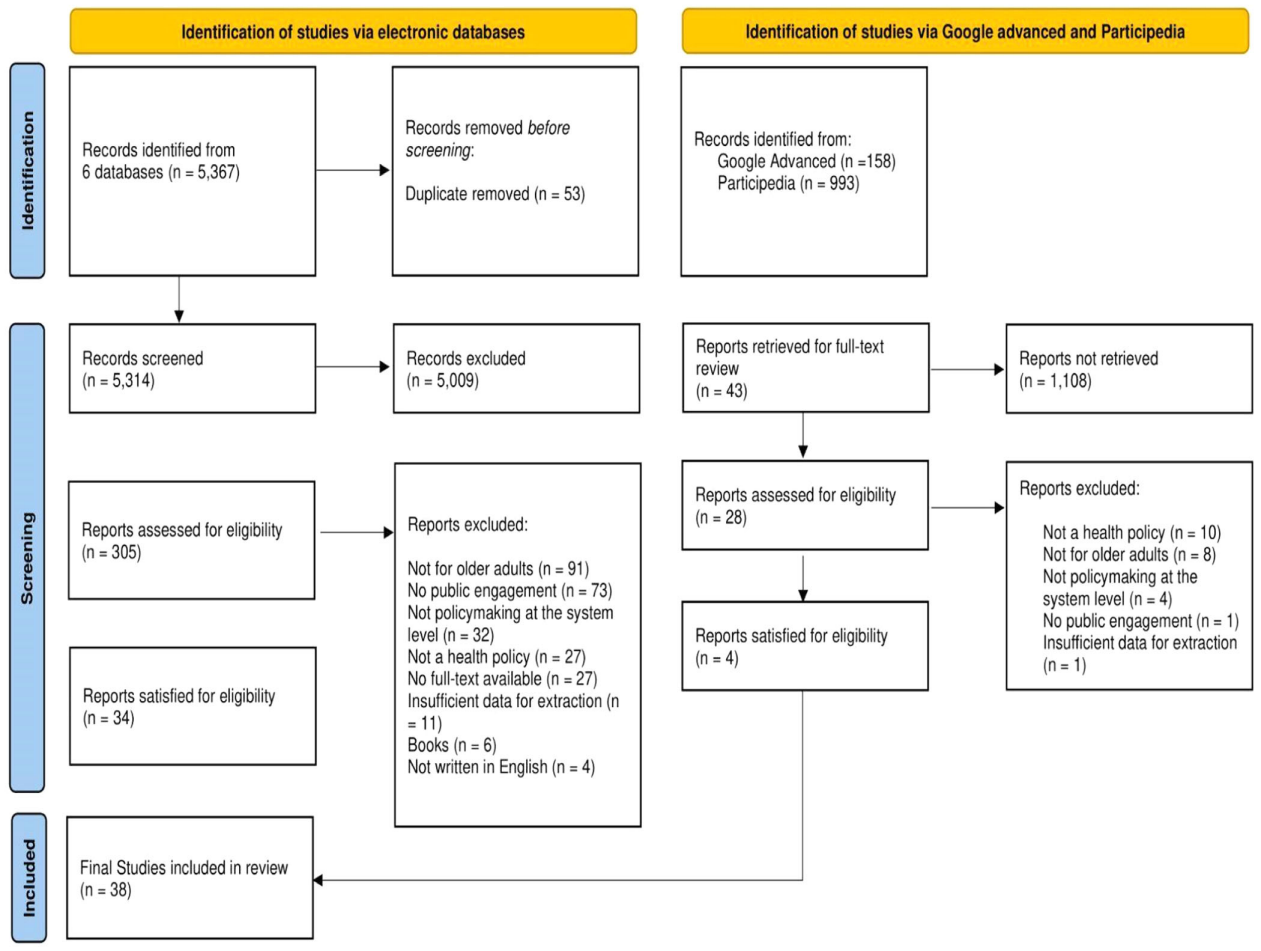
PRISMA flowchart.

The initial search of electronic databases, Google Advanced and Participedia yielded a total of 6518 articles for screening. After removing duplicates, 38 articles were eligible for inclusion (Figure 1).

### 3.1 Descriptive Information

Among the 38 included articles, 4 were published in the 1990s, 12 from 2000 to 2009, 14 between 2010 and 2019 and 8 from 2020 to present. Most described PE was carried out in Canada and the United States (*n* = 19), with some from Australia (*n* = 5), the United Kingdom (*n* = 5) and Ireland (*n* = 2). Fewer articles reported on PE in Israel, Poland, Spain, Thailand and internationally. The included articles cover a diverse range of policy‐making processes seeking PE input across different healthcare sectors and for different health conditions. These include long‐term care and home care (*n* = 10), cancer (*n* = 5) and mental health and neurological disorders (*n* = 4), among others. Characteristics of each included study are reported in Supporting Information S1: Appendix 2.

Among the included articles, 13 met all the criteria for methodological quality or the quality of grey information [17, 18]. Seven articles either failed to pass the first two screening questions of the MMAT tool or received a ‘can't tell’ response across all categories, indicating insufficient information for assessment. The remaining 18 articles showed varying degrees of criteria satisfaction. Common issues mostly arose due to inadequate detail for appraisal (e.g., insufficient data to assess the coherence between data collection, analysis and interpretation), resulting in a ‘can't tell’ response. However, as the methodological quality typically does not impact the inclusion of articles in a scoping review [10], we analysed all articles that met the inclusion criteria regardless of their appraisal results.

### 3.2 Links to Policy‐Making and Health System Arrangements Covered

More than half of the PE initiatives described in the included articles (*n* = 22) were designed to directly contribute to health policy‐making processes. These were typically funded by governments or government agencies to inform the policy‐making process at hand [20, 21, 22, 23, 24, 25, 26, 27, 28, 29, 30, 31, 32, 33, 34, 35, 36, 37, 38]. In some specific cases, these PE initiatives were required elements of the legislative process [39, 40, 41]. The other type of PE initiative identified in the articles aimed to inform policy‐making but without a direct link to a specific policy‐making process (*n* = 18). Some of these were initiated to draw the attention of policymakers and other key partners [23, 42, 43, 44]. Others were initiated within research projects [45, 46, 47, 48, 49, 50, 51] or did not explicitly report how the initiatives were related to a specific policy‐making process [23, 26, 42, 52, 53, 54, 55, 56, 57].

The majority of initiatives focused on health systems delivery (*n* = 23), concerning the design of care to meet consumers' needs [23, 26, 27, 29, 31, 32, 35, 38, 42, 43, 44, 48, 49, 52, 53, 56, 57], determining who should provide care and where [22, 24, 34, 42, 53] and the necessary supports for providing care [25, 30, 42, 50, 54]. Other initiatives centred on the governance of health systems (*n* = 8), including consumer and stakeholder participation in policy/organizational decisions (e.g., developing a national ageing strategy) [23, 27, 28, 36, 37, 50] or system monitoring [20, 26]. Financial aspects of health systems were addressed in some initiatives (*n* = 8), such as expanding the list of covered or reimbursed health services [40, 41, 46, 53, 57], decisions regarding who should pay for the reimbursement services or financing options [29, 51] and determining reimbursement rates [39].

Several initiatives were not focused on particular aspects of health systems but were rather broadly designed to gather general views on policy problems and or solutions (*n* = 6) [21, 23, 31, 33, 45, 47, 55].

#### 3.2.1 Engagement Participants, Approaches and Reported Impacts

Table 2 summarizes key data from all included articles, organizing them based on the type of engagement indicated in the far‐left column (e.g., ‘consult’, ‘deliberate’, ‘collaborate’ and ‘mixed’) [14]. Information is also reported about the participants in each PE initiative (e.g., older adults, family members of older adults, those representing or advocating for older adults and general public), the specific engagement methods used (e.g., survey, focus groups and citizen dialogues) and the reported impacts of each PE initiative. ‘Intrinsic’ impacts refer to the values of ensuring public inclusion, addressing the democratic deficit and fostering healthier democracy [15]. ‘Instrumental’ refers to improvements in policy‐making processes and outcomes such as enhancing policy responsiveness and policy buy‐in [15]. Lastly, ‘developmental’ refers to impacts on participants, including enhanced citizens' self‐worth and empowerment [15]. Each category of findings is discussed separately below.

**Table 2:** Detailed characteristics of all included studies grouped according to type of engagement.

Type of engagement	Author (year)	PE participants	PE methods^a^	Reported impacts
Consult	Aronson (1993)	Older adults only	Consultation meetings	Negative
	Ayalon (2021)	Older adults and others	Interviews	N/R
	King (2009)	Older adults only	Interviews	N/R
	McCormack (2000)	Older adults only	Community consultation	Negative
	van Riet‐Nales (2020)	N/R	Public consultation	Instrumental
	Steiner (2020)	Older adults and others	Community forums	N/R
	Participedia, #5549 (2023)	Older adults and others	Public/community forums	Instrumental
	Fraczkiewicz‐Wronka (2019)	Older adults and others	Senior council	N/R
	Chaufan (2012)	Older adults not targeted^b^	Congressional hearings	Negative, instrumental
	Bhavsar (2019)	Older adults not targeted	Public comments on government websites	N/R
	Minkler (2008)	Older adults not targeted	Focus groups, follow‐up focus groups, and town hall meetings	Instrumental, developmental
	Chappell (1997)	Older adults only	Focus groups, telephone interviews	Instrumental, developmental
	Cornes (2008)	Older adults only	Interviews, listening events, questionnaires	Instrumental
	Deber (1995)	Older adults and others	Public meetings, consultations, public conference	Instrumental, other (i.e., advancing the field of PE science)
	Donner (2015)	Older adults and others	Online stakeholder survey, public engagement meeting	Instrumental
	Manthorpe (2007)	Older adults and others	Individual interviews, nominal groups	N/R
	O'Shea (2006)	Older adults and others	Postal survey, regional seminar	N/R
Deliberate	Crotty (2020)	Older adults not targeted	Citizen's jury	Other (i.e., advancing the field of PE science)
	Baena‐Canada (2018)	Older adults and others	Citizen's jury	Instrumental
	Chuengsatiansup (2019)	Older adults not targeted	Citizen's jury	Developmental, instrumental
	Rychetnik (2014)	Older adults and others	Citizen's jury	Intrinsic
	Gibney (2019)	Older adults and others	Delphi panels^c^	Instrumental
	Participedia, #5084 (2023)	Older adults and others	Citizen's reference panel^d^	N/R
	Barnes (2005)	Older adults only	User panels^e^	Instrumental, intrinsic
	Lehoux (2018)	Older adults and others	Workshop, forum	N/R
Collaborate	Schichel (2020)	Older adults not targeted	Project partners^f^	N/R
	Norlander (2004)	Older adults not targeted	Community‐state partnership	Instrumental, developmental
Mixed: Multiple activities were organized around a specific engagement initiative, utilizing more than one type of activity.	Mattison (2020)	Older adults not targeted	Multiple activities were conducted such as citizen brief (share‐type), citizen's panel (deliberate‐type), and stakeholder meetings (collaborate‐type).	Instrumental, intrinsic
	Whitford (2006)	Older adults not targeted	Multiple activities were conducted for the ombudsman including share, consult, deliberate and collaborate‐type activities	Other (i.e., increased volunteerism is linked to higher levels of reported and investigated complaints)
	McWilliam (1997)	Older adults and others	Multiple activities were conducted such as focus groups, telephone surveys (consult‐type), the programme advisory committee, workshop (collaborate‐type)	Instrumental
	McKellar (2020)	Older adults and others	Multiple activities were conducted such as gallery walk (deliberate‐type) and focus group, working groups (collaborate‐type).	Instrumental
	Gong (2009)	Older adults and others	Multiple activities were conducted such as focus groups (consult‐type), stakeholder committees, stakeholder meetings (collaborate‐type)	Instrumental
	Extermann (2021)	Older adults not targeted	Multiple activities were conducted such as working group (consult‐type) and working group (collaborate‐type)	Instrumental
	Taylor (2012)	Older adults and others	Multiple activities were conducted such as e‐consultation group (share‐type), workshop, creative art sessions, working groups, community consultation (consult‐type)	Instrumental
	Province of New Brunswick (2017)	Older adults and others	Multiple activities were conducted such as online consultations (consult‐type) and council members (collaborate‐type)	N/R
	Woolsey (2004)	Older adults not targeted	Multiple activities were conducted such as educational sessions (share‐type), surveys and deliberate forums (consult‐type)	Developmental
Others: Included articles describe a compilation of separate and discrete PE initiatives.	Coleman (2002)	Older adults and others	Consult‐type activities include public forums, public hearings, public meetings, surveys, telephone hotline; Collaborate‐type activities include workgroups, task force, planning council.	Instrumental
	Miller (2012)	Older adults and others	Consult‐type activities include consumer advocates meeting with state administrators. Collaborate‐type activities include policy‐related panels such as advisory groups, taskforces, workgroups.	Intrinsic, instrumental

^a^ PE methods are as described in the articles, but additional details are provided for some methods to enhance understanding.

^b^ PE initiatives related to policies for older adults but did not have a specific focus or intentional inclusion of older adults as a distinct group of participants.

^c^ Delphi panel is a group of experts aiming to achieve consensus on specific issues. The consensus‐reaching process is characterized by iteration, anonymity and seeking stability in responses.

^d^ Citizen reference panel is a non‐compulsory public jury that offers policy advice. Similar to a citizen's jury, it utilizes various engagement tools, but they convene over an extended period (e.g., five 1‐day meetings over 18 months) [58].

^e^ User panel in this article refers to community‐based older adults who identified policy issues, discussed current policy and services and participated in deliberations with government officials.

^f^ Project partners in this article were represented in a management board (responsible for overseeing the project at large and making financial and organizational decisions) and in a project group responsible for execution and connecting with the communities.

### 3.3 Participants Recruited to PE Initiatives

The included articles revealed a diverse range of participants recruited for PE initiatives. Individual older adults were the most commonly recruited lay participant group (*n* = 26), followed by groups or organizations considered or claiming to represent and advocate for older adults (*n* = 16) and the general public (*n* = 11). Family members of older adults were less frequently engaged (*n* = 9). Older adults were engaged either as the sole participant group or alongside other participants. Six articles describe PE initiatives that exclusively recruited older adults [21, 28, 29, 37, 42, 52]. In 20 articles, older adults participated with others, such as family members, individuals from organizations that represent or advocate the rights of older adults, the general public, health professionals and academic researchers [20, 23, 24, 25, 27, 30, 31, 32, 33, 35, 36, 39, 46, 47, 48, 49, 50, 54, 56, 57]. Notably, in 11 articles, PE initiatives did not specifically target recruiting older adults [22, 26, 34, 38, 40, 41, 43, 45, 51, 53, 55], although some of these initiatives may have included older individuals. In these initiatives, the lay participants include the general public or interested individuals [40, 45, 51, 53], community volunteers [26], individuals with lived experience [34], patient/citizen advocates [38, 43, 55] and family members or informal caregivers of the elderly [22, 41]. The reasons for not engaging with older adults as participants were mostly not reported. Only one article mentioned practicality, in the context of recruiting stratified purposive samples for citizens' jury activities [22].

The age range for defining older adults varied across the PE initiatives. In close to two‐thirds of included articles (*n* = 24), explicit age ranges or criteria were seldom used. However, when indicated, the lowest age threshold for defining older adults was 50 years or older [25, 29, 46, 49, 50], while the oldest minimum age point used was 70 years or older [56]. Five articles did not explicitly define the age of older adults, as they aimed to include a diverse range of ages spanning different age groups (e.g., general public) [25, 45, 50, 51, 53].

Most included articles (*n* = 23) did not report considerations to involve socially disadvantaged populations during the design and implementation phases of PE initiatives [20, 23, 24, 26, 27, 28, 30, 33, 36, 37, 38, 39, 40, 41, 44, 46, 47, 48, 50, 52, 55, 56, 57]. In instances where PE organizers actively sought to ensure participant diversity (*n* = 15), a combination of criteria was often used in the design of PE initiatives. Commonly used criteria for promoting diversity included geographic location (e.g., individuals from remote communities with a population of less than 3000) [22, 25, 31, 42, 43, 45, 49], gender/sex [22, 29, 35, 43, 45, 51, 53] and race/ethnicity/culture/language [21, 25, 29, 31, 32, 45, 54]. Other criteria such as age [22, 35, 43, 45, 51, 53], socioeconomic status [35, 45, 51, 53], disability [25, 34, 35], education level [22, 35] and occupation [22, 35] were also considered.

Among those initiatives seeking participant diversity, about half (7 of 15) employed inclusion strategies, primarily focusing on participant recruitment. For instance, PE organizers collaborated with public organizations [25] or community partners [54] or utilized the services of third‐party companies to ensure the recruitment of the target population that balanced diverse characteristics associated with social marginalization [22, 45, 53]. Other strategies at the recruitment stage include employing a multi‐staged selection process [35] and providing easy‐to‐understand examples to help potential participants grasp the complex inclusion criteria [57]. A few articles reported inclusion strategies related to the implementation of engagement initiatives, such as conducting meetings in languages other than English [32, 54] and offering multiple participation channels (e.g., email and telephone) to accommodate elderly and sick participants [32]. Table 3 reports additional detail about the participants, the type of participant diversity sought, and the inclusion strategies described in PE initiatives.

**Table 3:** Participants, participants diversity criteria and inclusion strategies in PE initiatives.

	Participant diversity criteria	Inclusion strategy	Author (year)
Older adults only	Language, ethnic minorities	N/R	Aronson (1993)
	Ethnic minorities, sexual minorities and socially isolated	N/R	Cornes (2008)
	Geographic location	N/R	King (2009)
Older adults and others	Gender, age, socioeconomic status, disability, education level and special interest group affiliations	A multi‐staged selection process and a participants' registry were created	McWilliam (1997)
	Language	Recruitment through the project's community partners; meetings were conducted in different languages	Gong (2009)
	Geographic location, ethnic minorities, disability and with or without lived experience/care needs	Participants identified through the Healthcare Commission	Manthorpe (2007)
	Geographic location and ethnic minorities	N/R	Province of New Brunswick (2017)
	Geographic location	N/R	Participedia case #5084 (2023)
	Language	Multiple PE channels used to cater to different languages and to accommodate those who were unable to attend in‐person meetings	Participedia case #5549 (2023)
Older adults not specifically targeted or not identified	Geographic location, gender, age, ethnic minorities, socioeconomic status and with or without lived experience/care needs	A third‐party organization was used to support diverse recruitment	Wilson (2020)
	Disability	N/R	Minkler (2008)
	Geographic location, gender, age, education level and occupation	A polling organization was hired to recruit participants	Crotty (2020)
	Geographic location, gender, age, education level and occupation	A polling organization was hired to recruit participants.	Chuengsatiansup (2019)
	Geographic location, gender and age	N/R	Norlander (2004)
	Gender, age and socioeconomic status	N/R	Woolsey (2004)

### 3.4 PE Types and Methods

**Figure 2:**
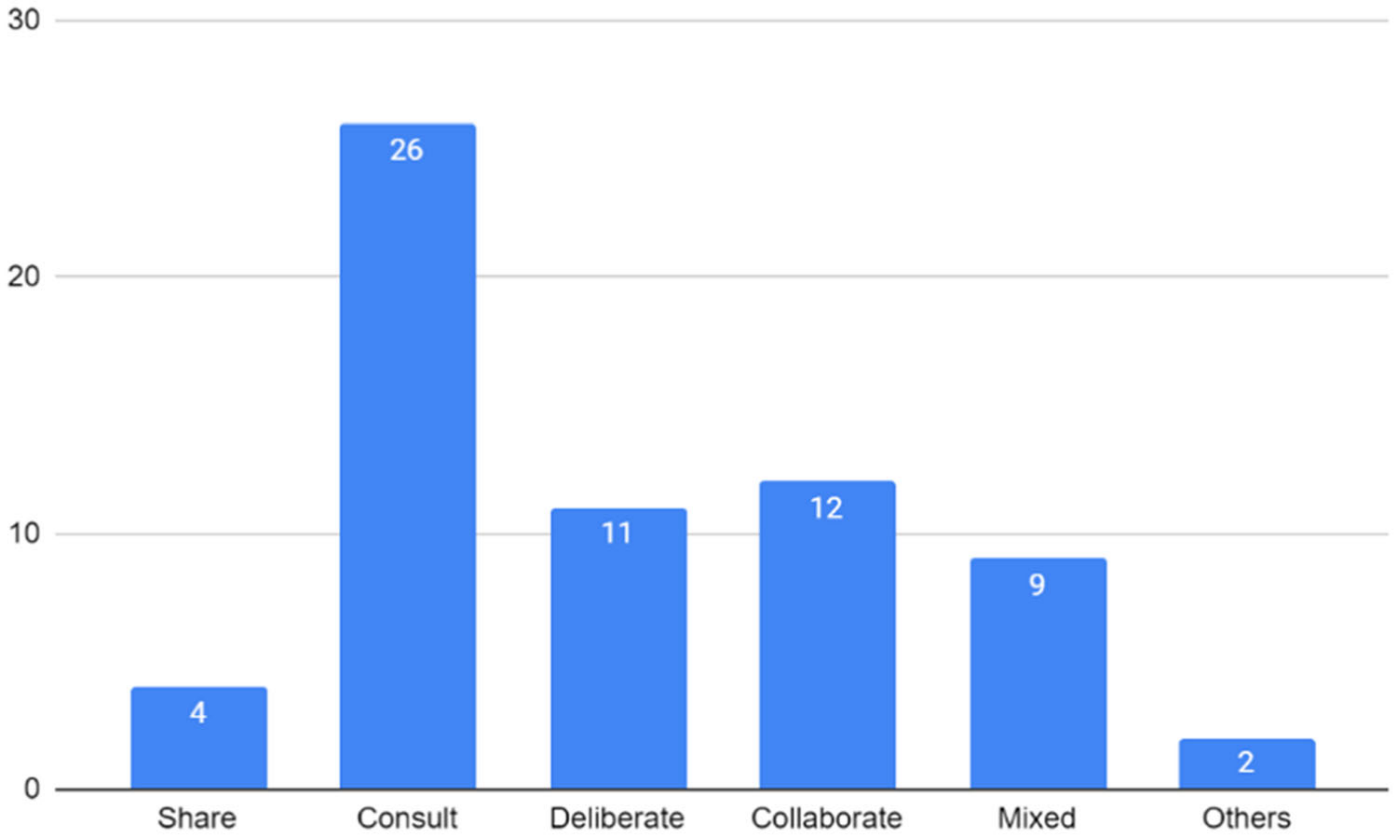
Number of PE initiatives by engagement type.

The PE initiatives described in the included studies employed various approaches, encompassing different types of engagement between PE organizers, participants and other stakeholders in the policy arena. These were categorized as ‘share’, ‘consult’, ‘deliberate’ and ‘collaborate’ [14] (see Figure 2 for the number of initiatives by engagement type).

The ‘share’ type of engagement, characterized by one‐way communication where PE organizers provide information to participants, was combined with other type(s) of activities in a small number of articles (*n* = 4) (i.e., classified as the ‘mixed’ type in Figure 2). Examples of such activities include the use of briefing documents [45] and an educational presentation [51], both conducted to inform subsequent engagements. Other examples involved educational sessions [26] and e‐consultation groups [30], both aimed at raising public awareness regarding policy issues.

‘Consult’‐type PE activities were the most commonly observed engagement approach (*n* = 26). These included consultation meetings [21, 28, 44], interviews [42, 56], surveys [33, 57], public forums [32, 48], public comments on government websites [40] and focus groups [34, 37]. Some articles reported single ‘consult’‐type engagement activities [21, 24, 28, 32, 33, 40, 41, 42, 44, 48, 56], while others described multiple ‘consult’‐type activities [23, 25, 29, 34, 37, 57].

‘Deliberate’ and ‘collaborate’ activities involve more interactive levels, where PE participants play a bigger role beyond mere information receivers and providers. These types were less commonly reported compared to the ‘consult’ type activities. Eleven articles described the ‘deliberate’‐type engagement. The most common form of ‘deliberate’‐type activities was the citizen's jury, where participants listen to expert presentations on background information, deliberate on policy options and formulate recommendations [22, 46, 50, 53]. Citizens' panels were also observed [20, 49, 52]. Twelve articles described ‘collaborate’‐type PE activities, which were often integrated into organizational governance structures. These activities involved collaboration between lay participants and other stakeholders, including professionals from different disciplines, industry stakeholders and academic researchers. Examples include project‐related panels such as project partners [38], advisory groups or committees [35, 54], councils [31], task forces [36] and working groups [27, 55].

Among the 38 included articles, nine reported a mix of different types of engagement within a single PE initiative. ‘Consult’‐ and ‘collaborate’‐type approaches were most commonly combined [31, 35, 54, 55]. ‘Share and consult’ [30, 51] and ‘deliberate and collaborate’ [27] were also combined, and, in some cases, more than two types of activities were combined [26, 45].

### 3.5 Reported PE Impacts

We examined the PE impacts as they were reported in the included articles, regardless of the quality of evidence supporting these impacts. Among the 38 included articles, 10 did not report on the impacts of PE initiatives [24, 25, 31, 40, 42, 47, 48, 49, 56, 57]. Of the remaining articles that reported impact information (*n* = 28), only a quarter (*n* = 7) met all the quality assessment criteria [34, 39, 50, 51, 59, 60, 61], another quarter (n = 8) met more than half [21, 30, 32, 33, 37, 52, 62] and the rest (*n* = 13) satisfied less than half of the criteria mostly due to insufficient information for assessment [23, 28, 29, 43, 44, 45]. This suggests that reported impacts may not fully reflect the real impacts of PE.

PE impacts were categorized into instrumental, intrinsic and developmental impacts [15]. Instrumental impacts were the most frequently mentioned, with 23 articles highlighting improvements to policy‐making processes and/or outcomes. Examples include the utilization of PE results as a project deliverable [20, 22, 27, 29, 30, 32, 33, 36, 46, 54, 55] or as integral parts of larger projects, such as informing issue refinement [35], shaping the next stage of a project [45] and facilitating dissemination [37]. Other examples included increased social awareness about policy issues and the need for policy change [34, 38, 39, 41, 44, 52], the adoption or citation of PE outcomes by reputable authorities [23, 37, 43], improved programme effectiveness [26] and serving as a counterpoint to powerful interest groups [39] (see Table 4 for details).

**Table 4:** Reported PE impacts.

Type of PE impacts	PE impact details	References
Instrumental (*n* = 23)	As a result of PE, policy outputs are developed (e.g., policy recommendations, tools, guidelines and frameworks)	[20, 22, 27, 29, 30, 32, 33, 36, 46, 54, 55]
	PE results partly contributed to a bigger project (e.g., issue refinement, PE findings informed a project's next stage and dissemination)	[41, 43, 51, 41, 43, 51]
	The actual adoption of the outcome of PE by a reputable authority (e.g., policymakers)	[29, 43, 49, 29, 43, 49]
	Increased social awareness about policy issues and a need for policy change against the status quo	[9, 40, 44, 45, 47, 50, 9, 40, 44, 45, 47, 50]
	Increased the programme effectiveness	[32]
	Served as a counterpoint to powerful interest group (i.e., industry)	[39]
Intrinsic (*n* = 4)	The best‐available research evidence was combined with citizens' values and preferences to inform the evidence brief	[51]
	Consumers and residents' advocates worked with legislators to ensure consumer representation in reimbursement reform	[45]
	The process of establishing the panels underscored the value of enabling frail older people to express their views and determine the issues and the agenda for action	[9]
	Through community juries, well‐informed public values and concerns were reflected in screening policies and programmes.	[50]
Developmental (*n* = 6)	Participants' knowledge about a programme/policy changed	[37, 51, 37, 51]
	Participants acquired advocacy skills and resources	[34, 43, 34, 43]
	Raised civic consciousness	[22]
Negative (*n* = 3)	Some doubt and scepticism arose about the motivation for PE and the possibility of PE making a difference	[21, 28, 21, 28]
	Caregivers' testimonies to Congressional committees legitimized the biomedical framework that deviates public support away from caregivers, ultimately benefiting biomedical scientists and depriving Alzheimer's disease sufferers of their humanity at the same time	[41]
Others (*n* = 3)	Advancing the field of PE science	[23, 53, 23, 53]
	Increased volunteerism may be linked to higher levels of reported and investigated complaints. However, increased monitoring activity does not dictate the quality of care for LTC residents	[32]
Not reported (*n* = 10)	N/A	[24, 25, 31, 40, 42, 47, 48, 54, 56, 57, 24, 25, 31, 40, 42, 47, 48, 54, 56, 57]

Several studies reported developmental impacts, such as increased participants' knowledge about specific programmes or policies [37, 51], the acquisition of advocacy skills and resources [34, 43] and the development of civic consciousness [22]. Intrinsic impacts were reported in a small number of studies. They emphasized the need for policy agendas and decisions to consider public values and concerns [50, 52]. Additionally, the importance of incorporating public values and preferences to ensure that policies or programmes are well‐informed was highlighted as a valuable source of evidence [39, 45]. Alongside these positive impacts, some articles discussed negative impacts, including doubt and scepticism concerning the motivation behind PE and the perceived effectiveness in influencing policy issues at hand [21, 28]. Unexpected consequences were noted in one study, where caregivers of patients with Alzheimer's disease participated in public hearings in Congressional committees that inadvertently legitimized the biomedical framework while undermining the humanity of patients and their caregivers [41]. A few articles documented additional impacts, including a connection between increased voluntary engagement and higher levels of reported and investigated complaints in policy programmes, suggesting increased monitoring activity [26] and the advancement of the science of PE [23, 53].

The sources of evidence for the reported impacts varied among the included articles. Primary sources included evaluation activities directly associated with PE initiatives, such as evaluation surveys [29, 50, 51, 52, 53, 54], voting outcomes [46], interviews [22, 39] and focus groups [22]. Participants' narratives were also utilized as a source of evidence [34, 37]. In six studies, the articles themselves focused on describing the processes and outcomes of PE activities (e.g., the development of a policy tool), serving as evidence of the instrumental impacts of the initiatives [20, 27, 31, 34, 35, 45]. Some articles relied on secondary sources by referencing external documents and reports that mentioned the impacts of their PE initiatives [26, 32, 34, 36, 37], while others reported impacts without specifying sources [21, 23, 28, 30, 33, 41, 43, 44, 55]. The variety of evidence sources for the reported impacts, combined with the mixed methodological quality of included articles, makes it difficult to assess the accuracy and reliability of the reported impacts.

## 4 Discussion

This scoping review examined the published and grey literature to determine how PE in health policy‐making for older adults has been conducted. Specifically, we described the participants engaged in these initiatives, the engagement approaches used and the reported impacts of these initiatives.

### 4.1 Older Adults' Participation and Participant Diversity

Our findings show that older adults have actively participated in a wide range of PE initiatives, either as the sole participant group or more frequently alongside other participants. Some PE initiatives that did not specifically target older adults as participants still involved individuals in the later stages of life. These results contribute to a more nuanced understanding of the participation of older adults in policy‐making. Previous studies have emphasized the barriers and challenges associated with engaging older adults leading to assumptions that older adults may be reluctant to participate in PE initiatives, or that they are less likely to be effectively involved [63]. These barriers and challenges include older adults' physical and cognitive frailty [64], ageist structures and attitudes in society [6, 65] and other practical barriers (e.g., geography and finances) [6, 64, 65]. Our findings challenge stereotypes that the frailty of older adults prevents them from understanding and reflecting on complex policy information [35, 45, 56]. These findings are significant because older adults, including those with frailty, express their desire to provide their input into policy‐making that matters to them [7, 63].

Our findings note a lack of reported efforts to capture and promote participant diversity across the PE initiatives, which may reflect the exclusion of socially and structurally marginalized populations and result in policies that inadvertently exacerbate this marginalization [63, 65]. Our older adult partners emphasized that the lack of diversity may create bias in the input provided by PE initiatives, possibly leading to policy decisions that favour those whose interests are already well‐represented in policy processes [6]. To actively seek diverse perspectives, relevant inclusion strategies need to be employed, tailored to the participants that PE initiatives aim to engage with, as the types of barriers and how they affect individuals' participation may vary depending on the target population's characteristics [65].

### 4.2 Engagement Methods to Promote Older Adults' Participation

We identified a diverse range of PE methods, ranging from one‐way communication types (i.e., ‘share’ and ‘consult’ type activities), to more interactive participation (i.e., ‘deliberate’‐ and ‘collaborate’‐type activities). Many typologies of PE suggest that PE impact increases as PE activities move along a continuum of enhanced collaboration and power‐sharing between participants and organizers, providing intuitive explanations on why lower levels of engagement may not deliver desired outcomes [66, 67, 68]. However, our findings suggest that the level of PE alone does not determine the impacts, especially in terms of positive and negative impacts. We found that negative impacts such as participants' doubt and scepticism regarding the true motives of PE [21, 28] and unexpected policy outcomes that negatively affected participants [41] were only observed in consultation‐type activities. In contrast, the articles that described share‐type activities combined with other types of engagement reported positive impacts only [26, 30, 45, 51], despite share‐type activities usually being considered a lower level of engagement. In this case, the share‐type activities served as capacity‐building exercises to inform subsequent engagements or as ways to raise public awareness about policy issues generally and were used in conjunction with higher‐level engagements. This aligns with our results demonstrating that PE initiatives that incorporated multiple activities reported positive impacts only [23, 29, 34, 37].

While the impacts reported in the articles do not fully capture the actual impacts of PE initiatives, our findings echo existing literature, suggesting that going beyond the notions of engagement types and levels and instead adopting creative methods and support mechanisms might more effectively engage older participants and generate meaningful impacts [5, 69]. When dealing with complex or technical topics, in particular, traditional engagement methods may not provide effective channels for communicating and gathering information from older adult participants [70]. Instead, various interactive formats or combinations of formats, which integrate the advantages of each tool, can be used to make engagement processes more accessible and inclusive for older adult participants with different preferences and abilities to participate, therefore encouraging their engagement [69, 71]. These findings are reinforced by our results demonstrating that PE initiatives reported positive impacts only when multiple engagement methods were used in combination.

Our older adult partners emphasized the importance of providing supporting infrastructure or programmes that accompany PE activities, such as capacity‐building, inclusive engagement opportunities that involve diverse participants and flexible timelines for participants to build trust, willingness and capacity to meaningfully participate and generate impacts. These suggestions are consistent with existing evidence that emphasizes the need for true partnerships that carefully plan for who and how people will be engaged to create positive impacts [64].

### 4.3 A Need to Systematically Evaluate PE Impact

Many of the included articles either did not report on the impacts of PE initiatives or reported impacts that were not attributed to a specific source (e.g., directly observed through process tracing or self‐reported through surveys or interviews). This lack of systematic evaluation of PE impacts is a notable gap in the PE literature [72]. Our older adult partners emphasized that without systematic evaluation, the reported impacts of PE initiatives may not fully encompass the diverse range of actual impacts, including positive or negative outcomes, short‐term or long‐term and tangible or intangible impacts. Current practices of only counting the immediate output of PE initiatives and not taking other types of impacts into account contrast with the desire of many PE participants and researchers to measure a wide range of PE impacts [73]. An active and comprehensive evaluation of PE not only demonstrates the return on investment for implementing these initiatives but can also guide and improve future practices [73]. Additionally, documenting the achievements of PE activities can help participants recognize the impact of their contributions, validating their past engagement and motivating them to participate in future initiatives [74]. Since each PE initiative is unique, PE organizers and researchers are encouraged to design their own evaluation frameworks by adapting existing instruments [75].

#### 4.3.1 Strengths and Limitations

This review engaged older adult partners in multiple stages of the study, which strengthened the relevance of the findings to one of the key audiences for our work. Another strength is that we were able to search the literature regarding policies for older adults, without prescribing a clear age cut‐off which acknowledges the reality and diversity of health policy‐making for older adults and the lack of specified age ranges for relevant policies. However, because there was no clear definition of ‘older adults’, this review relied upon authors' reporting on who the policies are for. Another limitation of this review is that many included articles did not have PE as the primary focus of the articles, which posed challenges when applying quality appraisal tools originally developed for scientific research to assess the descriptions of PE initiatives. The GRIPP2 reporting checklist assists with the quality assessment of public patient involvement (PPI) in research, regardless of whether the primary focus of a research study is on PPI or not [76]. A similar tool tailored to the policy‐making context would be useful for PE researchers in the field of policy‐making. Lastly, this review only included English language publications. This may have resulted in the exclusion of relevant articles contributing to the gap in our understanding of relevant PE initiatives in non‐English‐speaking jurisdictions, as well as the potential for limited applicability of our findings to these settings.

## 5 Conclusion

The papers included in this review demonstrate that older adults can participate in policy‐making processes designed for them. Despite ageist perceptions that their frailty precludes them from comprehending complex policy information and therefore participating in such initiatives, engagement of older adults can generate positive impacts, particularly when multiple engagement approaches are built into the design that emphasizes inclusive approaches tailored to the characteristics of the target population for the engagement.

## Author Contributions

**Jeonghwa You:** conceptualization, methodology, software, data curation, investigation, validation, formal analysis, visualization, project administration, writing–original draft, writing–review and editing. **Rebecca Ganann:** conceptualization, methodology, investigation, validation, funding acquisition, writing–review and editing. **Michael Wilson:** methodology, writing–review and editing, conceptualization, validation. **Soo Chan Carusone:** project administration, investigation, validation. **Maggie MacNeil:** investigation. **Carly Whitmore:** investigation. **Andrea Dafel:** investigation. **Roma Dhamanaskar:** investigation. **Eugenia Ling:** investigation. **Lance Dingman:** methodology, validation. **A. Tina Falbo:** methodology, validation. **Michael Kirk:** methodology, validation. **Joyce Luyckx:** methodology, validation. **Penelope Petrie:** methodology, validation. **Donna Weldon:** methodology, validation. **Katherine Boothe:** conceptualization, writing–review and editing. **Julia Abelson:** conceptualization, supervision, methodology, validation, writing–review and editing, investigation.

## Conflict of Interest

The authors declare no conflicts of interest.

## Supporting information

Supporting information.

## Data Availability

This scoping review synthesizes data from publicly available sources (e.g., published articles, reports and websites). Electronic databases searched include MEDLINE, CINAHL, Politics Collection, HealthStar, Social Science Citation Index and AgeLine, which can be accessed directly through their respective platforms. Additionally, Google Advanced and Participedia websites were used. The specific search strategies are available in Supporting Information S1: Appendix S1. For further inquiries regarding the data used in this scoping review, contact the corresponding author Jeonghwa You at youj25@mcmaster.ca.
