# Supplementary material for: Public Engagement in Health Policy‐Making for Older Adults: A Systematic Search and Scoping Review
Source: Health Expect. 2024 Aug 26;27(4):e70008. doi: 10.1111/hex.70008 (PMC11347750; doi:10.1111/hex.70008)
Supplement: Supplementary file 1 — Supporting information. [file HEX-27-e70008-s001.docx]

**Appendix 1. Search Strategies**

OVID Medline (search 2022-February-28)

| 1. exp Policy Making/  2. exp Health Policy/  3. (policy adj3 (making or development or health or healthcare or health care)).mp. [mp=title, abstract, original title, name of substance word, subject heading word, floating sub-heading word, keyword heading word, organism supplementary concept word, protocol supplementary concept word, rare disease supplementary concept word, unique identifier, synonyms]  4. 1 or 2 or 3  5. exp Community Participation/  6. exp Public Opinion/  7. exp Stakeholder Participation/  8. ((community or public or patient or citizen or user or advoca* or stakeholder) adj3 (engag* or involv* or participat* or consult*)).mp. [mp=title, abstract, original title, name of substance word, subject heading word, floating sub-heading word, keyword heading word, organism supplementary concept word, protocol supplementary concept word, rare disease supplementary concept word, unique identifier, synonyms]  9. 5 or 6 or 7 or 8  10. exp Aged/ or exp Middle Aged/  11. exp Aging/ or exp Healthy Aging/  12. exp Geriatrics/  13. (Senior or Older adult or Older people or Elderly).mp. [mp=title, abstract, original title, name of substance word, subject heading word, floating sub-heading word, keyword heading word, organism supplementary concept word, protocol supplementary concept word, rare disease supplementary concept word, unique identifier, synonyms]  14. gerontology.mp.  15. 10 or 11 or 12 or 13 or 14  16. 4 and 9 and 15 |
| --- |

Ovid Healthstar (search 2022-February-28)

| 1. exp Policy Making/ (28929)  2. exp Health Policy/ (119088)  3. (policy adj3 (making or development or health or healthcare or health care)).mp. [mp=title, abstract, original title, name of substance word, subject heading word, floating sub-heading word, keyword heading word, organism supplementary concept word, protocol supplementary concept word, rare disease supplementary concept word, unique identifier] (121442)  4. 1 or 2 or 3 (172780)  5. exp Community Participation/ (48937)  6. exp Public Opinion/ (19205)  7. exp Stakeholder Participation/ (3289)  8. ((community or public or patient or citizen or user or advoca* or stakeholder) adj3 (engag* or involv* or participat* or consult*)).mp. [mp=title, abstract, original title, name of substance word, subject heading word, floating sub-heading word, keyword heading word, organism supplementary concept word, protocol supplementary concept word, rare disease supplementary concept word, unique identifier] (100161)  9. 5 or 6 or 7 or 8 (119133)  10. exp Aged/ or exp Middle Aged/ (4286543)  11. exp Aging/ or exp Healthy Aging/ (188208)  12. exp Geriatrics/ (31027)  13. (Senior or Older adult or Older people or Elderly).mp. [mp=title, abstract, original title, name of substance word, subject heading word, floating sub-heading word, keyword heading word, organism supplementary concept word, protocol supplementary concept word, rare disease supplementary concept word, unique identifier] (285876)  14. gerontology.mp. (4012)  15. 10 or 11 or 12 or 13 or 14 (4420517)  16. 4 and 9 and 15 (1199) |
| --- |

ProQuest: Politics Collection (search 2022-February-28)

| ((MAINSUBJECT.EXACT.EXPLODE("Health Policy") OR MAINSUBJECT.EXACT.EXPLODE("Policy Making")) OR ("policy" NEAR/3 ("making" OR "development" OR "health" OR "healthcare" OR "health care"))) AND (MAINSUBJECT.EXACT.EXPLODE("Citizen Participation") OR noft(("community" OR "public" OR "patient*" OR "citizen*" OR "user*" OR "advoca*" OR "stakeholder*") NEAR/3 ("engag*" OR "involv*" OR "participat*" OR "consult*"))) AND (((MAINSUBJECT.EXACT.EXPLODE("Geriatrics") OR MAINSUBJECT.EXACT.EXPLODE("Long Term Care")) OR (MAINSUBJECT.EXACT.EXPLODE("Middle Aged Adults") OR MAINSUBJECT.EXACT("Aging"))) OR (aged OR aging OR senior* OR older adult* OR older people OR elderly)) |
| --- |

Web of Science: Science Citation Collection (search 2022-February-28)

| 1. TI=(policy near/3 ("making" or "development" or "health" or "healthcare" or "health care")) OR AB=(policy near/3 ("making" or "development" or "health" or "healthcare" or "health care"))  2. policymaking (All Fields)  3. #1 OR #2  4. (TI=((community or public or patient* or citizen* or user* or advoca* or stakeholder*) NEAR/3 (engag* or involv* or participat* or consult*) )) OR AB=((community or public or patient* or citizen* or user* or advoca* or stakeholder*) adj3 (engag* or involv* or participat* or consult*) )  5. #3 AND #4 |
| --- |

EBSCOHOST AgeLine (search 2022-February-28)

| S1. DE "Policy Making"  S2. policy N3 (development or making or health or healthcare or health care)  S3. S1 OR S2  S4. (community or public or patient or citizen or user or advoca* or stakeholder) N3 (engag* or involv* or participat* or consult*)  S5. S3 AND S4  S6. DE "Older Adults"  S7. DE "Aging" OR DE "Active Aging" OR DE "Aging in Place" OR DE "Biological Aging" OR DE "Healthy Aging"  S8. senior* or older adult* or elderly* or geriatric or aged  S9. S6 OR S7 OR S8  S10. S5 AND S9 |
| --- |

EBSCOHOST CINAHL (search 2022-February-28)

| S1. (MH "Policy Making") OR (MH "Health Policy+")  S2. policy N3 (making or development or health or healthcare or health care)  S3. S1 OR S2  S4. (MH "Stakeholder Participation") OR (MH "Political Participation+")  S5. TX (community or public or patient* or citizen* or user* or advoca* or stakeholder*) N3 (engag* or involv* or participat* or consult*)  S6. S4 OR S5  S7. S3 AND S6  S8. (MH "Aged+") OR (MH "Middle Age") OR (MH "Aging+")  S9. (MH "Geriatrics") OR "gerontology"  S10. "Senior or Older adult or Older people or Elderly"  S11. S8 OR S9 OR S10  S12. S3 AND S6 AND S11 |
| --- |

Participedia (search 2022-March-7) and Google Advanced (search 2022-April-18)

In conducting searches on Participedia, only keywords related to the concept 3 ‘older adults’ were used, considering the website’s focus on public engagement in policymaking. For Google Advanced searches (language: English; site or domain: .gov and .org), the three concepts below were combined using “AND”, while all key words within each concept were combined using “OR”.

| Concept 1: Health Policymaking | Concept 2: Public Engagement | Concept 3: Older adults |
| --- | --- | --- |
| Policy*  Health*  Long-term care | (public OR patient* OR citizen* OR stakeholder* OR advoca* OR community OR user* OR input) AND (engag* OR involv* OR participat* OR consult*) | Senior*  Older adult*  Elder*  Age* |

**Appendix 2. Characteristics of included articles**

| Author, year | Country | PE Objectives | Healthcare sector | Links to a policy-making | Health-system arrangement types |
| --- | --- | --- | --- | --- | --- |
| Mattison, 2020 | Canada | To identify citizens’ values and preferences for how to enhance equitable access to assistive technologies and to engage policymakers, stakeholders, and researchers in deliberations to spark action. | N/R | N | Others (Broadly defined topics) |
| Whitford, 2006 | USA | To assist LTC residents and their families within obtaining quality care | Long-term care/ nursing home | Y | Governance, Delivery |
| Minkler, 2008 | US | To develop, implement, and evaluate an individual and community empowerment and policy change intervention known as the Social Action Group (SAG) program. | Long-term care/ nursing home | Y | Delivery |
| McWilliam, 1997 | Canada | To evaluate and compare models of community-based primary care designed to promote and support the independence of seniors with chronic medical problems. | Primary care: community-based | Y | Delivery |
| McKellar, 2020 | Australia | To develop a framework to provide a way forward for South Australian older persons’ mental health services after The Oakden Report. | Mental health (Alzheimer’s disease) | Y | Governance, Delivery |
| McCormack, 2000 | Australia | To undertake a community consultation with older Australians to determine what they saw as the needs of older people, and how these matters might be addressed | N/R | Y | Governance |
| Crotty, 2020 | Australia | To explore the general public’s views on issues involved in the allocation of rehabilitation resources for residents of care facilities | Rehabilitation services: ppl living in residential care | N | Financial, Delivery |
| Coleman, 2002 | US | To pursue long-term care reforms in the context of tight budget constraints and growing concern about health-related expenditures. | Long-term care/ nursing home | Y | Governance |
| Chappell, 1997 | Canada | To identify seniors' views of drug reimbursement policies or their confidence in sources other than physicians for prescription medicines. | Multiple: Medications for stomach ulcers (H2 blockers); angina/heart attack/heart failure (nitrates); arthritis/joint pain (NSAIDS) | Y | Governance |
| Baena-Canada, 2018 | Spain | To know if women, when better informed via the information provided during the citizen jury process, are able to answer the question of whether the Andalusian Public Health System (SSPA) must continue offering screening mammography to women aged 50-69 | Mammographies | N | Financial |
| Gibney, 2019 | Ireland | To measure consensus on a set of key positive aging indicators for Ireland using the Delphi technique | N/R | Y | Governance |
| Miller, 2012 | USA | To influence Medicaid nursing home reimbursement policy. | Long-term care/ nursing home | Y | Financial |
| Gong, 2009 | USA | To identify health risks and develop intervention programs to improve health and safety through partnerships between in-home care workers and their clients (consumers) in Alameda County, California. | Home care | N | Delivery |
| Extermann, 2021 | International | To define the top priorities for the advancement of geriatric oncology worldwide | Cancer | N | Others (Broadly defined topics) |
| Aronson, 1993 | Canada | To gain the community input and advice into the design of long-term care services | Long-term care/ nursing home | Y | Others (Broadly defined topics) |
| Ayalon, 2021 | Israel | To evaluate an Israeli training program designed for caregivers of older adults. | Long-term care/ nursing home | N | Delivery |
| Bhavsar, 2019 | USA | To meet a legal requirement for public engagement on proposed regulations | End of life care (Advanced care) | Y | Financial |
| Chaufan, 2012 | United States | To obtain information regarding the dimensions of the problem and efforts to address Alzheimer’s disease | Mental health (Alzheimer’s disease) | Y | Financial |
| Chuengsatiansup, 2019 | Thailand | (1) To learn how the “citizens’ jury” as a deliberative forum can be used as a model for public engagement, (2) To learn about the public’s view on long-term care for elders as well as on its roles in health policy, and (3) To develop skills and strengthen the organization’s capacity on new forms of public participation | Long-term care/ nursing home | Y | Financial, Delivery |
| Cornes, 2008 | UK | To set standards for fair, high-quality, integrated services for older people in England | N/R | Y | Delivery |
| Deber, 1995 | Canada | (1) To improve long-term care policy decisions; (2) To promote a sense of empowerment among individuals by involving them in policymaking. | Long-term care/ nursing home | Y | Governance, Delivery, Others (Broadly defined topics) |
| Fraczkiewicz-Wronka, 2019 | Poland | To serve older persons by representing their interests within the municipality. Seniors’ councils gained a legal basis for their existence in 2013. | N/R | Y | Delivery |
| King, 2009 | UK | To influence the development of appropriate health and social care service provision for older people. | N/R | N | Delivery |
| Lehoux, 2018 | Canada | To explore the perceptions and preferences of potential users of assistive robots | Assistive robot | N | Others (Broadly defined topics) |
| Manthorpe, 2007 | UK | To evaluate the impact of the National Service Framework for Older People (NSFOP) on the experiences and expectations of older people | N/R | Y | Delivery |
| Schichel, 2020 | The Euregion Meuse-Rhine consisting of five regions with three language areas; from Germany, Belgium, the Netherlands | To address the limited use of cross-border exchange of health policies and best practices in Belgium, Germany and the Netherlands | Mental health (dementia, late-life depression) | Y | Delivery |
| Steiner, 2020 | Australia | To co-design a model of care for a new multidisciplinary memory clinic | Mental health (dementia) | N | Delivery |
| Taylor, 2012 | UK | To develop the Northern Ireland Single Assessment Tool (NISAT) for the health and social care of older people. | N/R | Y | Delivery |
| Province of New Brunswick, 2017 | Canada | To develop an ageing strategy that ensures a more responsive system where seniors can remain independent and engaged in their communities for as long as possible. | N/R | Y | Delivery, Others (Broadly defined topics) |
| Participedia, #5084, n.d. | Canada | (1) To elicit citizen values about breast cancer screening; and, (2) To explore policy options to support citizens in making an informed choice regarding mammography. | Mammography | N | Delivery |
| Participedia, #5549, n.d. | Canada | To better assess the specific needs of the elderly with very complex illnesses, in hope of being able to improve services for their specific, challenging needs | N/R | Y | Delivery |
| Donner, 2015 | Canada | To solicit input from a broad range of stakeholders, asking respondents about successes and challenges in the home and community care sector | Home care | Y | Others (Broadly defined topics) |
| Barnes, 2005 | UK | To promote the practices of deliberative democracy, where citizens, politicians and ‘experts ’ all have equal opportunity to pursue their arguments and challenge others, and where the intentions of all are focused on achieving the best possible policy outcome, rather than pursuing self interest. | N/R | N | Delivery |
| Norlander, 2004 | United States | To promote public policy that would advance improvements in end of life care in Minnesota by forming a state commission involving public and private representation. | End of life care (Advanced care) | N | Delivery |
| O'Shea, 2006 | Ireland | To evaluate the impact of a health promotion strategy for older people on stakeholders | Multiple: Cancer and heart disease | N | Financial, Delivery |
| Rychetnik, 2014 | Australia | To determine the priorities, values and concerns regarding prostate-specific antigen screening among men aged 50-70 who we fully informed about the reasons for and against screening | Cancer | N | Governance, Delivery |
| van Riet-Nales, 2020 | International (Europe) | To gain attention and feedback (either supportive or with a proposal for revision) on the pharmaceutical development of medicines for use in the older population. | Medicines (pharmaceuticals) | N | Delivery |
| Woolsey, 2004 | US | To determine whether and how participants’ Medicare reform preferences change as the result of involvement in an educational and deliberative forum | US Medicare | N | Financial |
